# Activation of LXRɑ/β by cholesterol in malignant ascites promotes chemoresistance in ovarian cancer

**DOI:** 10.1186/s12885-018-5152-5

**Published:** 2018-12-10

**Authors:** Soochi Kim, Maria Lee, Danny N. Dhanasekaran, Yong Sang Song

**Affiliations:** 1 Seoul National University Hospital Biomedical Research Institute, Seoul, 03080 Republic of Korea; 2 Cancer Research Institute, Seoul National University College of Medicine, Seoul, 03080 Republic of Korea; 3 Department of Obstetrics and Gynecology, Seoul National University College of Medicine, Seoul, 03080 Republic of Korea; 4 Stephenson Cancer Center, university of Oklahoma Health Sciences Center, Oklahoma City, OK 73012 USA; 5 Interdisciplinary Program in Cancer Biology, Seoul National University College of Medicine, Seoul, 03080 Republic of Korea; 6 Biomodulation, Department of Agricultural Biotechnology, Seoul National University, Seoul, 03080 Republic of Korea

**Keywords:** Ascites, Cholesterol, Chemoresistance, LXRα/β,, Ovarian cancer

## Abstract

**Background:**

The purpose of this study was to investigate the role of malignant ascites tumor microenvironment in ovarian cancer progression and chemoresistance.

**Methods:**

A total of 45 patients with ovarian cancer and three benign ascites were collected at the time of clinical intervention. Ascites cholesterol levels were quantitated using cholesterol quantitation kit and recurrence free survival (RFS) of ovarian cancer patients were collected. The sensitivity of ovarian cancer cells to cisplatin (CDDP) and paclitaxel (PAC) were assessed by viability assay, flow cytometry and protein expression. Receiver operating characteristics (ROC) curve and Youden index analysis were applied to calculate the optimal cut-off values for ascites cholesterol. Kaplan-Meier curve were applied to compare RFS between high and low ascites cholesterol levels in ovarian cancer patients.

**Results:**

Here we show that cholesterol is elevated in malignant ascites and modulates the sensitivity of ovarian cancer cells to CDDP and PAC by upregulating the expression of drug efflux pump proteins, ABCG2 and MDR1, together with upregulation of LXRɑ/β, the cholesterol receptor. Transfection of LXRɑ/β siRNA inhibited cholesterol-induced chemoresistance and upregulation of MDR1. In addition, the cholesterol level in malignant ascites was negatively correlated with number of CDDP-induced apoptotic cell death, but not with that of PAC-induced apoptotic cell death. Cholesterol depletion by methyl beta cyclodextrin (MβCD) inhibited malignant ascites-induced chemoresistance to CDDP and upregulation of MDR1 and LXRɑ/β. For patients with ovarian cancer, high cholesterol level in malignant ascites correlated with short RFS.

**Conclusions:**

High cholesterol in malignant ascites contributes to poor prognosis in ovarian cancer patients, partly by contributing to multidrug resistance through upregulation of MDR1 via activation of LXRɑ/β.

**Electronic supplementary material:**

The online version of this article (10.1186/s12885-018-5152-5) contains supplementary material, which is available to authorized users.

## Background

Ovarian cancer is the most lethal gynecologic cancer resulting from the late diagnosis and eventual chemoresistance followed by frequent recurrence [1]. Standard treatment of ovarian cancer includes, maximal cytoreductive surgery and adjuvant taxane and platinum based chemotherapy. Initial response rate is very high, 70–80% including 40 to 50% complete response, but majority of patients relapse within 2 years with subsequent resistance to chemotherapy [2]. Despite recent advances in anticancer therapy of ovarian cancer, ovarian cancer survival remains poor and our understanding of ovarian cancer progression including chemoresistance is still very limited.

There is a growing evidence indicating the importance of tumor microenvironment in ovarian cancer progression, especially chemoresistance [3]. The aberrant accumulation of fluid in the peritoneal cavity called ascites formation occurs in more than one third of ovarian cancer patients and in almost all recurrent cases [2, 4]. Recent progress in deciphering the cellular and acellular components of ascites has shown that ascites serves as an important tumor microenvironment enriched in pro-tumorigenic signals that contribute to enhanced invasiveness and chemoresistance [5, 6]. Moreover, the presence of ascites correlates with the disease stage and poor prognosis in ovarian cancer patients [7, 8]. It is critical to elucidate the mechanism of chemoresistance to improve the survival rate for ovarian cancer.

Previous studies have shown that cholesterol is significantly elevated in ascites and could be used as a marker for malignant ascites [9, 10]. Additionally cholesterol has been shown to be involved in the regulation of drug response in a number of cancer models [11, 12]. Cholesterol is required for cellular signals including proliferation. However, an excess cellular cholesterol is toxic and thus the levels of cholesterol are tightly regulated and coupled to pathways that enable the removal of cholesterol [13, 14]. The transcription factors of the liver X receptor (LXR) family provide a feed-forward regulatory system for the elimination of excess cholesterol [15]. Recently, LXRα was reported to exhibit oncogenic properties in gastric cancer cells [16]. There is no study about the relationship between the cholesterol in malignant ascites and chemoresistance in ovarian cancer. Our objective is to know the effect of malignant ascites cholesterol on chemoresistance and explore the mechanism of chemoresistance by the cholesterol in ascites. Our present study reveals that the cholesterol is elevated in malignant ascites derived from ovarian cancer patients and correlates with the chemoresistance and reduced recurrence free survival (RFS). In addition, cholesterol enhances chemoresistance to cisplatin (CDDP) and paclitaxel (PAC) via Liver x receptor α/β (LXRα/β) mediated induction of multidrug resistance protein expression, MDR1 protein in vitro.

## Methods

### Cell culture, clinical samples and reagents

PA-1, OVCAR-3, and SKOV-3 used in this study were obtained from the American Type Culture Collection (Rockville, MD). With the exception of PA-1, these cell lines were grown in RPMI1640 (WelGENE, Seoul, Korea). PA-1 was cultured in MEM (WelGENE, Seoul Korea). All culture media were supplemented with 10% FBS (Gibco-BRL, Gaithersberg, MD), and 100 μg/mL penicillin-streptomycin (P/S) (Invitrogen, Carlsbard, CA).

Ascites from 30 serous, 6 mucinous, 6 clear and 3 mixed ovarian cancer patients and three benign ascites were collected at the time of clinical intervention at the Seoul National University Hospital (Seoul, Korea). This study was approved by the Institutional Review Board (IRB) at Seoul national University Hospital (Registration number: 1409–1540-616), and prior written and informed consent was obtained from every patient. Ascites were centrifuged at 2500 rpm for 20 min. The acellular fractions were filtered (70 μm), aliquoted and stored at − 80 °C to minimize freeze-thaw.

### Cell viability assay

Cell viabilities were evaluated by the Thiazolyl blue tetrazolium bromide (MTT) assay. Cells were seeded in 96-well plate. After overnight incubation, an increasing cisplatin (CDDP) concentration from 0 to 20 μM were adjusted to a final volume of 100 μl/well for indicated time. The cells were incubated with 50 μL MTT (2 mg/ml, 3 h, 37 °C) in 5% CO_2_ in humidified atmosphere and subsequently solubilized in DMSO 100 μl/well. The optical density at 540 nm was determined using an enzyme linked immunosorbent assay reader.

### Cell death analysis

Using flow cytometry analysis, apoptotic cell death was determined via Annexin-V and PI staining (BD Pharmingen, CA) according to the manufacturer’s protocol.

### Western blotting

Protein lysates were prepared as described previously [11]. In brief, after cell extraction, proteins were separated by SDS/PAGE (6–15% gel, depending on specific protein assessed) followed by electrotransfer onto nitrocellulose membranes and probed with the indicated antibodies.

### Reagents and antibodies

Stock solutions of cisplatin (Enzo life science) and paclitaxel (LC Laboratories,) were prepared in Dimethylformamide; 0.001% and water soluble cholesterol and methyl β-cyclodextrin (MβCD) (Sigma-Aldrich, St. Louis, MO) were prepared in DEPC water and used at the final concentration indicated. MTT was from Amresco (Olon, OH). Antibodies to ABCG2 (monoclonal, 1:1000 dilution), PARP (polyclonal, 1:1000 dilution), LXRα/β (monoclonal, 1:1000 dilution) and Lamin B (polyclonal, 1:5000 dilution) were purchased from Santa Cruz Biotechnology (Santa Cruz, CA). Antibodies to MDR1 (monoclonal, 1:1000 dilution), pan Cadherin (polyclonal, 1:1000 dilution) were purchased from Cell Signaling (Danvers, MA). GAPDH (monoclonal, 1:5000 dilution) (AB frontier) and α tubulin (monoclonal, 1:5000 dilution) (Santa Cruz, CA) were used as a loading control.

### Nuclear and cytoplasmic protein extraction

Using NE-PER nuclear and cytoplasmic extraction reagents from Pierce Biotechnology (Rockford, USA), cytoplasmic and nuclear proteins were separated and extracted from cancer cells, according to the manufacturer’s protocol.

### Membrane and cytoplasmic protein extraction

Using Membrane Protein Extraction Kit from BioVision (Milpitas, CA), membrane and cytoplasmic proteins were separated and extracted from cancer cells, according to the manufacturer’s protocol.

### Small interfering RNA transfection

The siRNA-targeting LXRα (sc-38,828) purchased from Santa Cruz and scrambled RNA (mBio Tech, Gyeonggido, Korea) was used as a negative control. Cells were transfected using RNAi reagent obtained from Invitrogen (Carlsbad, CA), according to the manufacturer’s protocols.

### Ascites cholesterol quantitation

Ascites cholesterol level were quantitated using cholesterol quantitation kit (Sigma-Aldrich, St. Louis, MO). Ascites were diluted 5–10% to that of final volume, and the cholesterol level were quantitated according to the manufacturer’s protocol. Serum ascites cholesterol gradient (SACG) was calculated as Serum Cholesterol – Ascites Cholesterol.

### Cignal reporter assay

Cells were plated on a 96-well plate. Cignal LXR reporter (Qiagen) 100 ng was transfected using Lipofectamine 2000™ reagent obtained from Invitrogen (Carlsbad, CA), according to the manufacturer’s protocols. At 24 h post-transfection, cells were treated either with or without cholesterol. At 6 h after cholesterol treatment, cells were harvested and reporter assays were performed using a dual luciferase reporter assay system (Promega) according to the manufacturer’s instructions. *Renilla* and firefly luciferase activities were measured using Luminescence Counter VICTOR™ Light (Perkin Elmer, NJ).

### Primary cell isolation from ovarian cancer patient derived ascites

Primary cells were isolated and cultured as previously described [17]. In brief, ascites derived from ovarian cancer patients were centrifuged at 2500 rpm for 10 min. Cells were re-suspended in PBS and cells were isolated using Ficoll-Paque™-PREMIUM centrifugation at 2500 rpm for 30 min. The collected cells were cultured in the complete culture medium.

### Statistical analysis

Data were presented as mean ± SEM of triplicate experiments. One-way ANOVA and, when appropriate, Student’s t-test were used for statistical analyses. Significant difference among experimental groups was analyzed by Scheffe’s post hoc test. Receiver operating characteristics (ROC) curve and Youden index analysis were performed to determine the optimal cut-off values for ascites cholesterol. All analyses were conducted using IBM SPSS statistics 21 (SPSS Inc., Chicago, IL). *P* values of < 0.05 were considered statistically significant.

## Results

### Response of ovarian cancer cell lines to CDDP and PAC correlates with ABCG2 and MDR1 protein expression

**Fig. 1:**
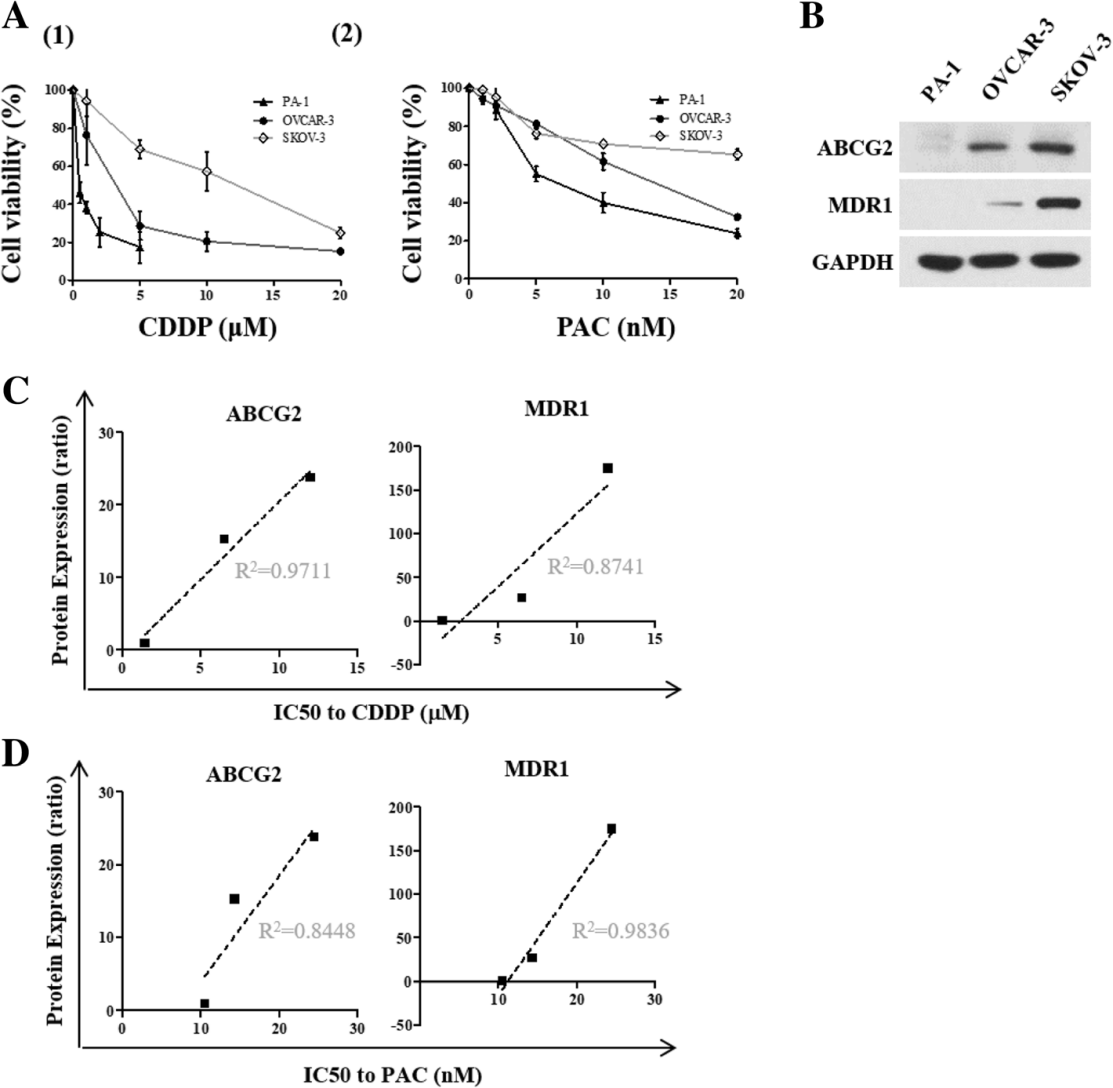
ABCG2 and MDR1 protein expression is correlated with resistance to cisplatin and paclitaxel in ovarian cancer cells. (**A1** and **A2**) Comparison of ovarian cancer cell viability following CDDP and PAC treatment for 48 h. **a** and **b**) The indicated concentrations of CDDP and PAC were treated to three ovarian cancer cell lines for 48 h. Cell viability was determined by MTT assay. **b** ABCG2 and MDR1 protein expression was measured by Western blot. **c-d**. Protein expression levels were quantitated by densitometry and normalized to GAPDH. The ratio of densities was calculated using PA-1 as control. The correlation between CDDP IC50 and ABCG2/MDR1 protein expression, PAC IC50 and ABCG2/MDR1 protein expression were determined by simple linear regression analysis. The correlation coefficient square (R^2^) was determined by Pearson’s correlation coefficient test

CDDP and PAC treatment causes a dose- and time- dependent decrease in cell viability of three ovarian cancer cell lines, PA-1, OVCAR-3 and SKOV-3 cells and CDDP IC50 was calculated to be 1.4 μM, 6.5 μM and 12 μM respectively and PAC IC50 was calculated to be 10.5 nM, 14.3 nM and 24.5 nM respectively (Fig. 1a). In ovarian cancer, expression of ATP-binding cassette transporter (ABC transporter) proteins including ABCG2 and MDR1 are closely related with drug resistance [18, 19]. More than half the family members of ABC transporter confer drug resistance [20]. Of those ABCG2 and MDR1 are modulated by cholesterol and cholesterol synthesis inhibitor, statins [11, 12, 21, 22]. Indeed, ABCG2 and MDR1 protein expression were relatively high in SKOV-3 compared to PA-1 and OVCAR-3 (Fig. 1b) and IC50 to CDDP and PAC were directly correlated with ABCG2 and MDR1 protein expression (Fig. 1c and d).

### Cholesterol enhances chemoresistance to CDDP and PAC through reduction in apoptosis

**Fig. 2:**
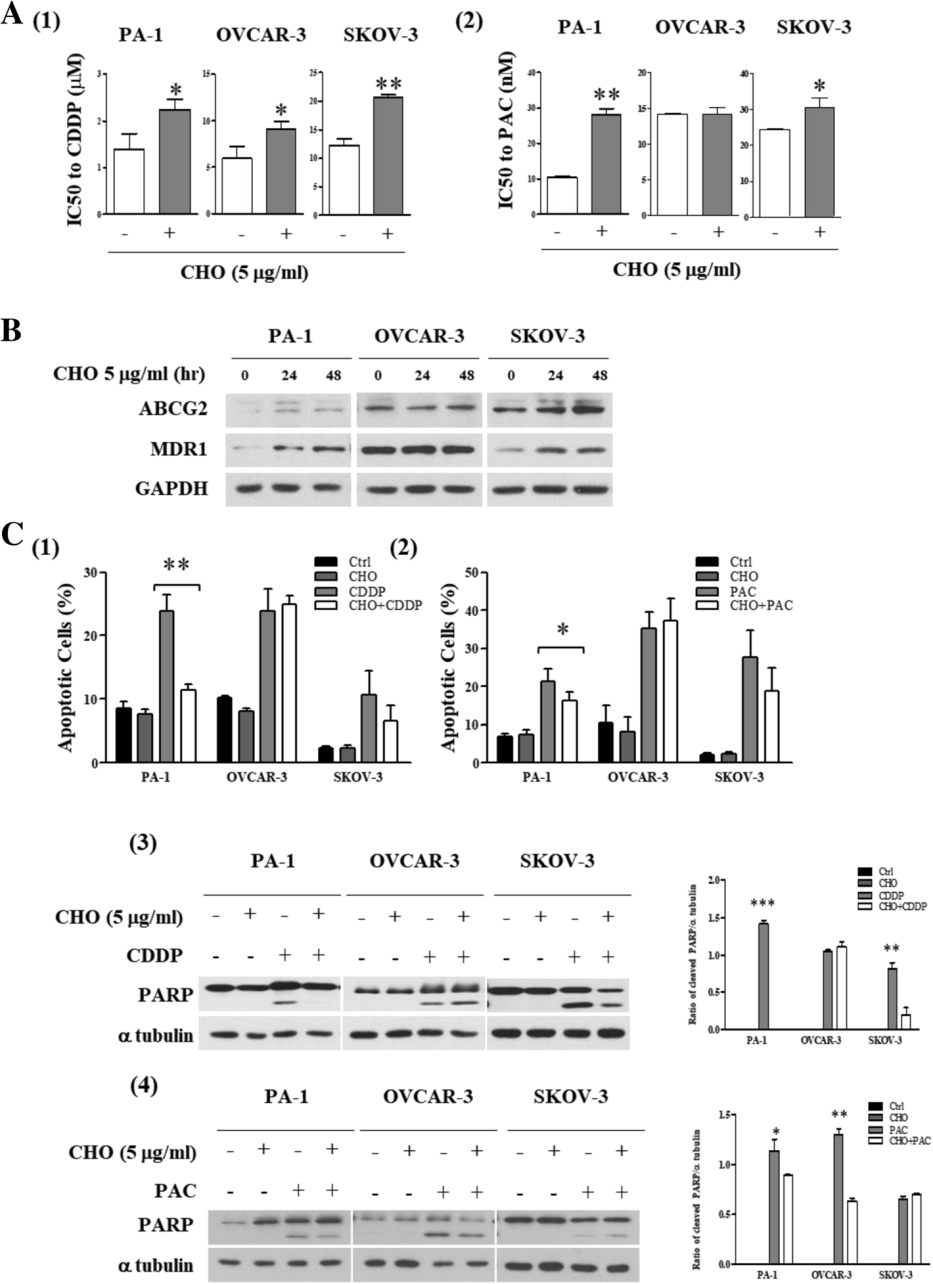
Increase of CDDP and PAC resistance after a 24 h exposure of cholesterol in ovarian cancer cell lines. Ovarian cancer cell lines were pretreated with 5 μg/ml cholesterol for 24 h. (**A1**) Comparison of IC50 to CDDP and (**B2**) PAC before and after cholesterol treatment, determined from the data by MTT assay. **b** Expression of ABCG2 and MDR1 protein 24–48 h after 5 μg/ml cholesterol treatment by Western blot. (**C1** and **C2**) Number of apoptotic cells induced by CDDP and PAC, determined by Annexin V/PI staining. (**C3** and **C4**) Protein expression of cleaved PARP by Western blot and densitometry analysis of cleaved PARP relative to α tubulin. Significant differences are indicated as follows. *P < 0.05, **P < 0.01, ***P < 0.001

Circulating free cholesterol levels are tightly regulated and cholesterol have been shown to be involved in the regulation of various membrane proteins, including ABCG2 [11, 12]. Moreover, lowering cholesterol synthesis with HMG-CoA reductase inhibitor reduced ovarian cancer risk [23–25]. To explore the role of cholesterol in ovarian cancer chemoresistance, we applied various concentration of water soluble cholesterol (CHO) containing media (0, 5, 10, 20 μg/ml). Ovarian cancer cells were pre-treated with an indicated cholesterol concentration for 24 h and cell viability were determined using MTT assay (Additional file 1: Figure S1A). To ensure that cholesterol loading vehicle, methyl-β-cyclodextrin (MβCD), depleting cholesterol in solution has no toxicity, at the indicated concentration of MβCD after 24 h treatment (Additional file 1: Figure S1B). For all subsequent experiments, 5 μg/ml cholesterol was used with no significant effect on cell viability of ovarian cancer cells. Cholesterol pretreatment significantly increased IC50 to CDDP and PAC in PA-1 and SKOV-3 cells, not in OVCAR-3 cells (Fig. 2a and Additional file 1: Figure S2). This was further confirmed with ascites derived ovarian cancer cells, A8, A39 and A53 at passage between 14 and 18 (Additional file 1: Figure S2). Accordingly, cholesterol treatment increased the expression of ABCG2 and MDR1 protein in PA-1 and SKOV-3 cells but to a less extent in OVCAR-3 cells (Fig. 2b). Notably, cholesterol pretreatment significantly reduced both CDDP and PAC induced apoptotic cell death only in PA-1, verified by Annexin V/PI staining Fig. 2 C1 and C2. Cholesterol pretreatment significantly reduced CDDP mediated cleaved PARP expression only in PA-1 and SKOV-3 and PAC mediated cleaved PARP expression only in PA-1 and OVCAR-3, analyzed by Western blot (Fig. 2 C3 and C4). Altogether, cholesterol enhances chemoresistance to CDDP and PAC in ovarian cancer cells. PA-1 cells were chosen for further evaluation of molecular mechanisms of cholesterol-induced drug resistance.

### LXRα/β mediates cholesterol-induced chemoresistance in ovarian cancer

**Fig. 3:**
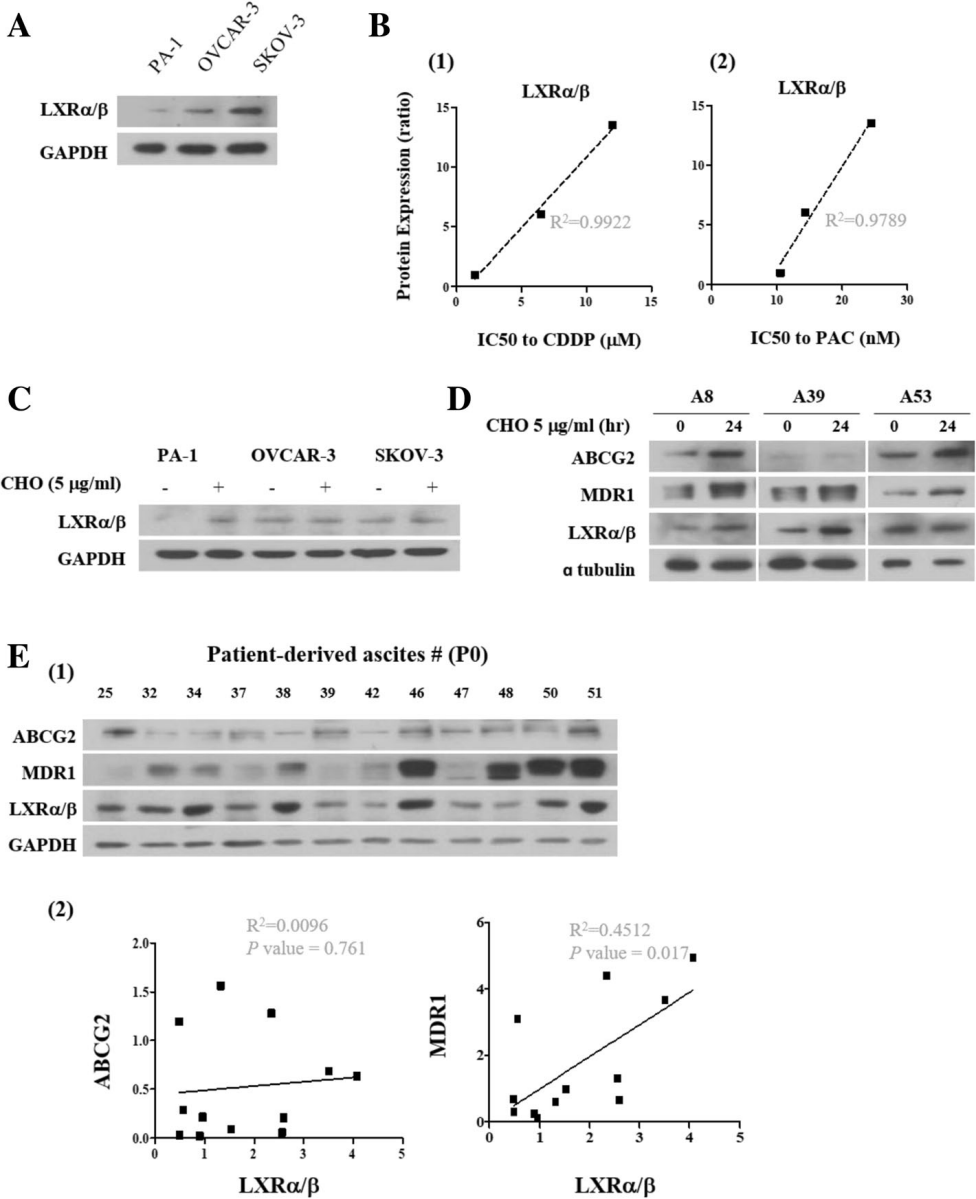
LXRα/β protein expression is correlated with resistance in ovarian cancer cells. **a** LXRα/β protein expression was measured by Western blot. **(B1** and **B2)** Protein expression levels were quantitated by densitometry and normalized to GAPDH. The ratio of densities was calculated using PA-1 as control. The correlation between CDDP IC50 and LXRα/β protein expression, PAC IC50 and LXRα/β protein expression was determined by simple linear regression analysis. **c** Expression of LXRα/β protein after cholesterol (5 μg/ml) treatment measured by Western blot. **d** Expression of ABCG2, MDR1 and LXRα/β protein was measured by Western blot with cholesterol (5 μg/ml) treatment in ascites derived ovarian cancer cells. (**E1** and **E2**) LXRα/β protein expression levels were measured by Western blot in primary cancer cells isolated from ovarian cancer patient derived ascites. Protein expression levels were quantitated by densitometry and normalized to GAPDH. The correlation coefficient square (R^2^) was determined by Pearson’s correlation coefficient test

Cholesterol is an essential components of mammalian cell membranes, which generates a semipermeable barrier between cellular compartments and modulates the functions of membrane proteins [26]. Circulating cholesterol levels are tightly regulated as excess cholesterol is toxic [14]. The liver x receptor (LXR) family, provide a feed-forward regulatory system for the elimination of excess cholesterol, positively regulating the expression of genes encoding lipid transport proteins [13, 15]. Moreover, previous report suggests cholesterol as an essential modulator of the ABCG2 and MDR1 functions [22]. Interestingly, LXRα/β protein expression was relatively high in SKOV-3 compared to PA-1 and OVCAR-3 (Fig. 3a) and IC50 to CDDP and PAC were directly correlated with LXRα/β protein expression (Fig. 3b). Cholesterol treatment increased the expression of LXRα/β protein in PA-1 and ascites derived ovarian cancer cells, A8, A39 and A53 (Fig. 3c and d). We also found that MDR1 but not ABCG2 protein expression was positively correlated with LXRα/β in primary cells isolated from ovarian cancer patient derived ascites at passage 0 (Additional file 1: Figure S3E). Information of primary cancer cells are shown in Additional file 2: Table S1.

**Fig. 4:**
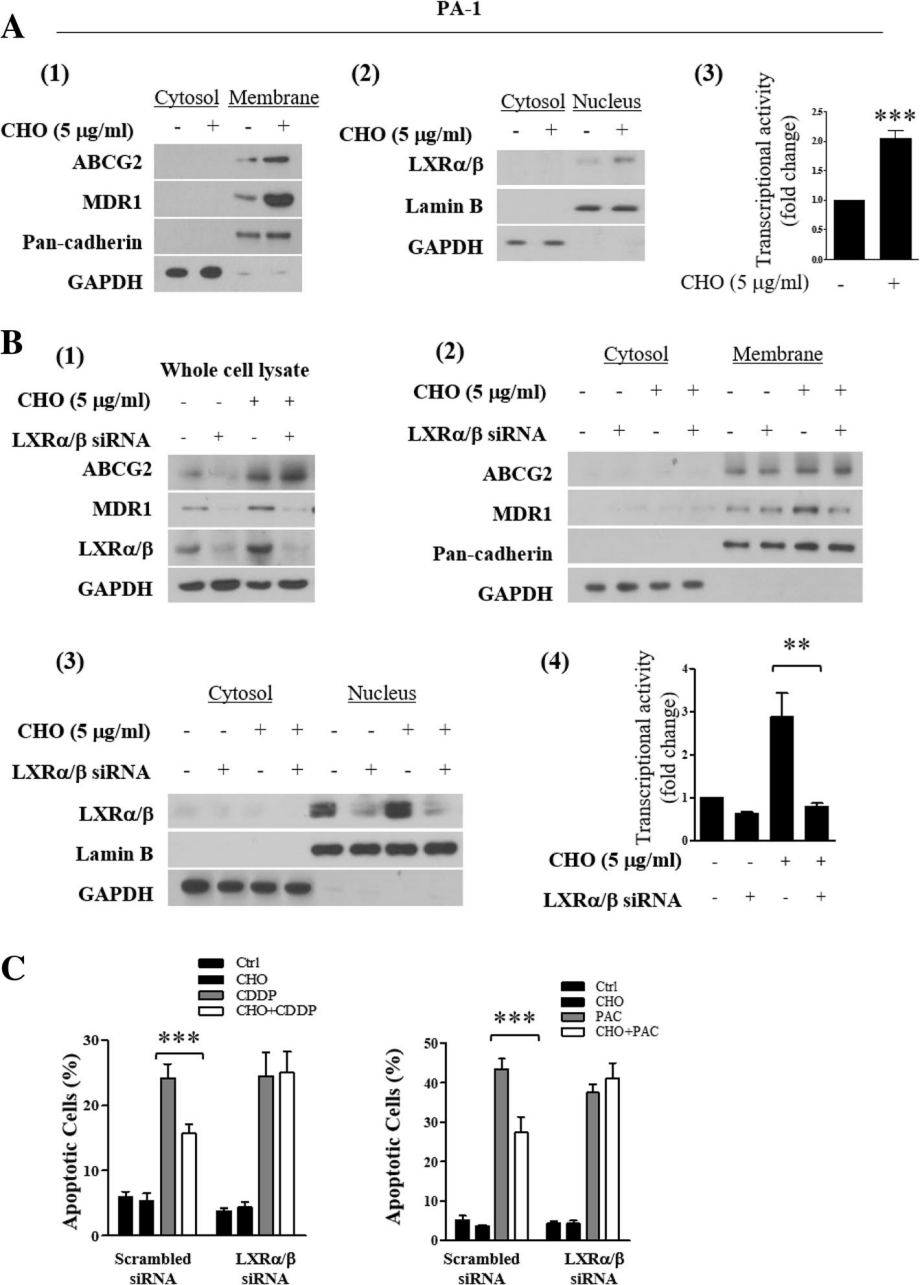
Silencing of LXRα/β inhibits cholesterol-induced chemoresistance. PA-1 cell line was pretreated with 5 μg/ml cholesterol for 24 h. **(A1)** Western blot of ABCG2, MDR1 in cytosol and membrane fraction. (**A2**) Western blot of LXRα/β in cytosol and nuclear fraction. (**A3**) Transcriptional activity of LXRα/β determined by luciferase reporter gene assay. **b-c** Effect of LXRα/β silencing by siRNA transfection on cholesterol-induced chemoresistance. PA-1 cells were transfected with LXRα/β targeted siRNA or scrambled siRNA (80 nM) as a negative control. Then PA-1 cells were treated with CDDP or PAC in the presence or absence of cholesterol. (**B1**) Expression of ABCG2, MDR1 and LXRα/β protein using whole cell lysates, (**B2**) cytosol and membrane fractions, (**B3**) cytosol and nucleus. (**B4**) Transcriptional activity of LXRα/β. **©** CDDP- and PAC- induced apoptotic cell death determined by Annexin V/PI staining. Significant differences are indicated as follows. **P < 0.01, ***P < 0.001

Using PA-1 cell line, we further evaluated the role of LXRα/β protein and cholesterol in chemoresistance. Treatment of cholesterol for 24 h increased membrane expression of both ABCG2 and MDR1 and nuclear translocation and transcriptional activity of LXRα/β in PA-1 (Fig. 4a). Silencing LXRα/β with siRNA impaired cholesterol–induced MDR1 overexpression but did not affect ABCG2 overexpression, confirmed by Western blotting using whole cell lysate, membrane and nuclear fraction (Fig. 4b). Also, silencing LXRα/β significantly decreased cholesterol induced chemoresistance (Fig. 4c).

### Clinical implication of high cholesterol in ascites and LXRα/β in ovarian cancer development and cancer progression

**Fig. 5:**
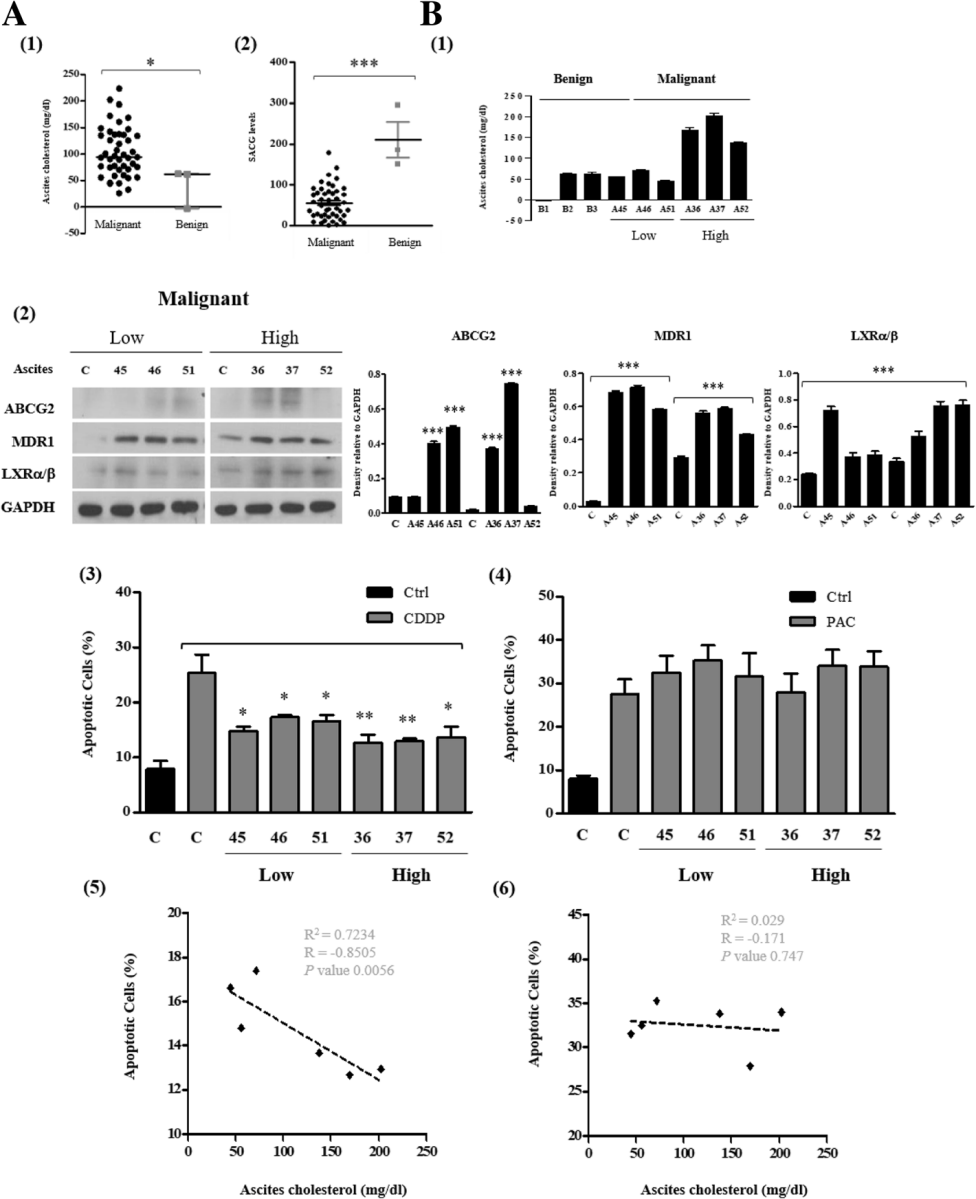
Cholesterol in ovarian cancer patient derived ascites promotes chemoresistance. (**A1**) Comparison of cholesterol concentrations between malignant and benign ascites. (**A2**) Comparison of serum ascites cholesterol gradient (SACG) between malignant and benign ascites. SACG was calculated by ascites cholesterol subtracted from serum cholesterol. **b** Malignant ascites were divided into two groups, low versus high groups, using an optimal cut-off value calculated from a ROC curve and Youden index analysis. (**B1**) Cholesterol concentration of benign and malignant ascites in low vs high group. PA-1 cell line was treated with indicated malignant ascites for 24 h. (**B2**) Western blot and densitometry analysis of ABCG2, MDR1 and LXRα/β protein in whole cell lysates. (C = control media without malignant ascites). (**B3** and **B4**) CDDP and PAC induced apoptotic cell death determined by Annexin V/PI staining 24 h after treatment with each respective malignant ascites. (C = control media without malignant ascites, black box indicate control without CDDP/PAC treatment) (**B5** and **B6**) Correlation between cholesterol levels in malignant ascites and relative ratio of number of CDDP- and PAC- induced apoptotic cell death. The correlation coefficient square (R^2^) was determined by Pearson’s correlation coefficient test. Significant differences are indicated as follows. *P < 0.05, **P < 0.01, ***P < 0.001

The cholesterol level is significantly higher in malignant ascites, which can be used to discriminate malignant ascites from benign ascites [9, 10]. We postulated that cholesterol in malignant ascites from patients with advanced ovarian cancer modulate response to CDDP and PAC, causing therapy failure in ovarian cancer patients with ascites. Forty-five ascites were collected from ovarian cancer patients and three from benign conditions. The patient characteristics of malignant group are shown in Additional file 3: Table S2 and non-malignant group in Additional file 4: Table S3. Both total and free cholesterol levels in ascites were quantitated using cholesterol quantitation kit and serum cholesterol levels were simultaneously checked. The cholesterol level was significantly higher in malignant ascites than in peritoneal fluid from patients with benign ovarian cyst (Fig. 5 A1). The serum ascites cholesterol gradient (SACG, calculated as serum cholesterol subtracted by ascites cholesterol) levels were significantly lower in ovarian cancer patients than in benign patients (Fig. 5 A2). To determine the role of cholesterol in malignant ascites, patients were divided into two groups depending on the levels of cholesterol with high cholesterol group equal to or greater than 70 mg/dL in ascites and low cholesterol group less than 70 mg/dL. The optimal cut-off value 70 mg/dL was derived from a ROC curves and a Youden index analysis (data not shown). The ascites cholesterol levels from three representing patients of each group are shown in Fig. 5 B1. Treatment with ascites of low and high cholesterol group increased the expression of MDR1 and LXRα/β expression in PA-1 cell lysate (Fig. 5 B2). Interestingly, ascites pretreatment significantly reduced CDDP and CDDP in combination with PAC induced apoptotic cell death but did not when PAC were treated alone in PA-1 cells (Fig. 5 B3, B4 and Additional file 1: Figure S3). In parallel, cholesterol levels in ascites were inversely correlated with the number of CDDP induced apoptotic cell death and marginally when CDDP were treated in combination with PAC but not with that of PAC induced apoptotic cell death in PA-1 cells (Fig. 5 B5, B6 and Additional file 1: Figure S3).

**Fig. 6:**
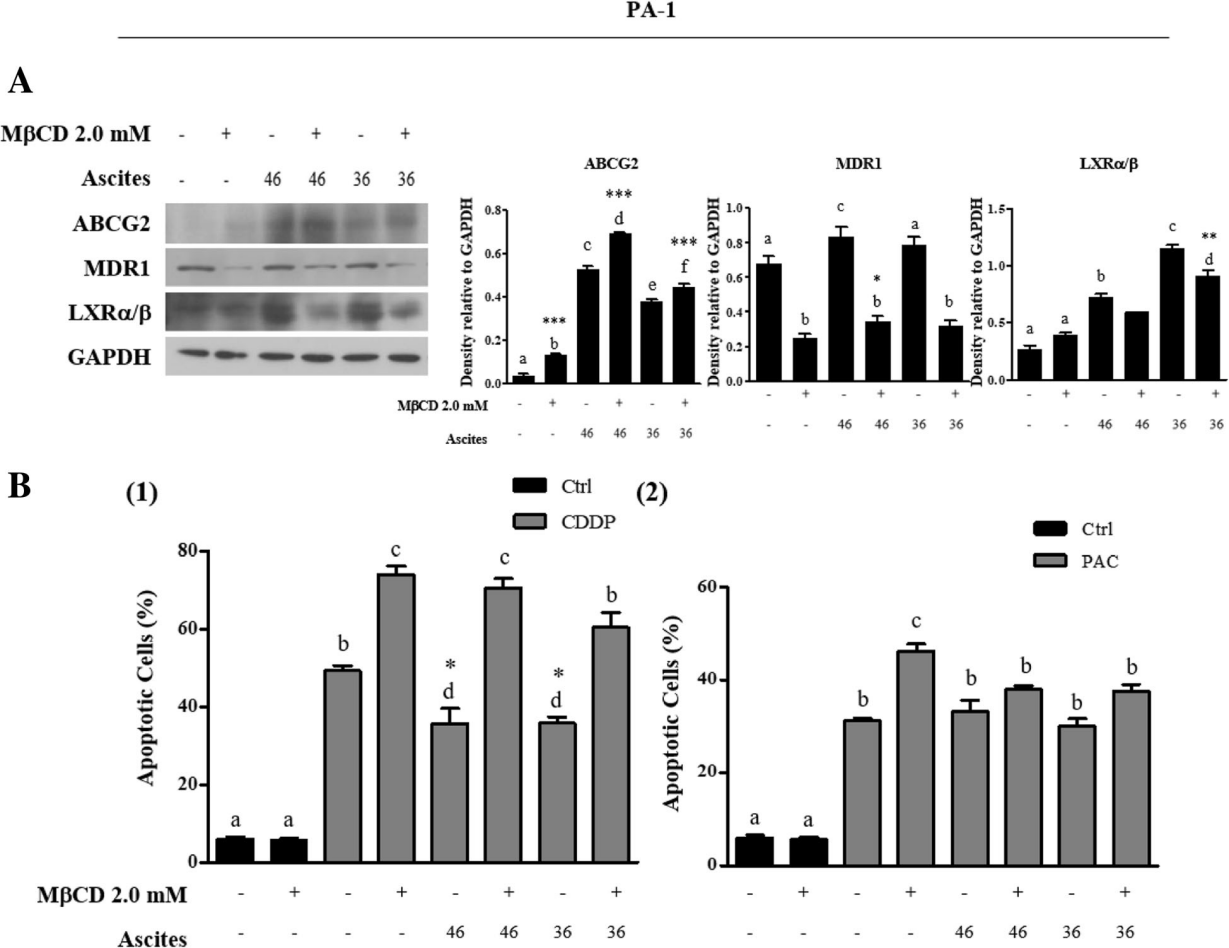
Cholesterol depletion from malignant ascites by methyl β cyclodextrin (MβCD) inhibits malignant ascites-induced chemoresistance against CDDP. **a** Western blot of ABCG2, MDR1 and LXRα/β in whole cell lysates 24 h after co-treatment with malignant ascites and MβCD (cholesterol depleting agent in solution). (**B1** and **B2**) CDDP and PAC induced apoptotic cell death after co-treatment with malignant ascites and MβCD. Apoptotic cells determined by Annexin V/PI staining. (Black box indicate control without CDDP/PAC treatment). Significant differences are indicated as follows. *P < 0.05, **P < 0.01, ***P < 0.001 a, b, c, d, e, f indicates homogeneous subsets, using one-way ANOVA followed by Scheffe’s post hoc test

To further confirm that the chemoresistance is acquired due to the enriched cholesterol microenvironment, cholesterol was depleted with methyl beta cyclodextrin (MβCD) [27]. Co-treatment of malignant ascites with MβCD for 24 h significantly reduced MDR1 and LXRα/β expression (Fig. 6a). More importantly, co-treatment of malignant ascites with MβCD significantly decreased CDDP induced apoptotic cell death but did not PAC-induced apoptotic cell death (Fig. 6 B1 and B2). These data support an important role of the cholesterol in malignant ascites induced resistance in ovarian cancer.

**Fig. 7:**
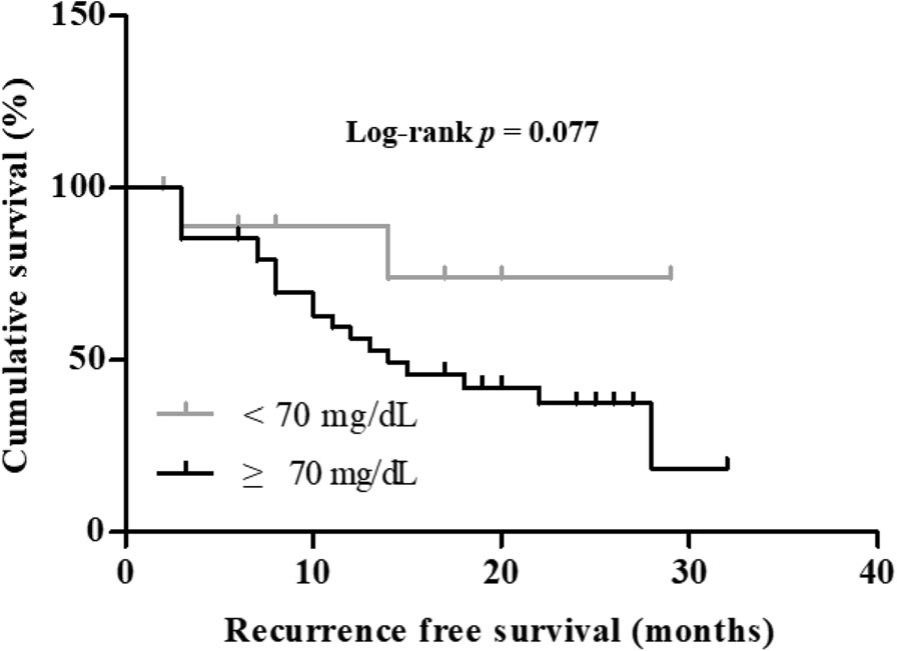
High concentration of cholesterol in malignant ascites correlates with shorter recurrence free survival (RFS). Comparison of Kaplan-Meier curve for RFS between high and low cholesterol levels in ascites, more than 70 mg/dL vs equal to or less than 70 mg/dL, in ovarian cancer patients

To know the clinical implication of elevated cholesterol in malignant ascites, recurrence free survival (RFS) duration was compared between ovarian cancer patients with ascites cholesterol levels < 70 mg/dL and ≥ 70 mg/dL. The mean values of RFS were 21 and 14 months in ascites cholesterol levels < 70 mg/dL and ≥ 70 mg/dL groups, showing marginal significance (*p =* 0.077) (Fig. 7). Together, these results suggest that elevated cholesterol in ascites may be associated with the chemoresistance in ovarian cancer patients. Collectively, we show that cholesterol activate LXRα/β in ovarian cancer cells, causing the chemoresistance to CDDP through the upregulation of MDR1 expression.

## Discussion

Acquired chemoresistance is the major reason for the failure of ovarian cancer treatment. Both presence of malignant ascites and acquired chemoresistance have been associated with reduced survival of ovarian cancer patients [28, 29]. In this study, we demonstrate that ovarian cancer cells exhibit increased multidrug resistance, such as CDDP and PAC, when exposed to cholesterol alone or to patient derived malignant ascites. To our knowledge, our work is the first to directly demonstrate acquired chemoresistance by cholesterol in malignant ascites.

As opposed to serum, ascites being a proximal fluid to cancer cells themselves provide local tumor microenvironment rich in many cancer-associated soluble factors [30]. These soluble factors are either cause of or result of underlying disease. Indeed, the presence of ascites correlates with reduced quality of life and a poor prognosis in ovarian cancer [8, 31]. Among soluble factors accumulated in malignant ascites, we found that cholesterol is significantly higher in malignant ascites from patients with ovarian cancer than peritoneal fluid from patients with benign cyst (Fig. 5 A1). This is consistent with the previous reports by Rana et al. [10]. Moreover, cholesterol levels in malignant ascites were inversely correlated with CDDP induced apoptotic cell death but not with PAC (Fig. 5 B5 and B6). Additionally, high cholesterol in malignant ascites is associated with shorter RFS (Fig. 7). Our results suggest that cholesterol in malignant ascites may play an important role in acquired chemoresistance to CDDP in ovarian cancer.

Acquired chemoresistance by malignant ascites was first reported in ovarian cancer-bearing mice model through increased expression and function of ABC transporters [32]. However, a detailed mechanism regulating ABC transporters in malignant ascites tumor microenvironment is unclear [32, 33]. Cholesterol, a steroidal lipid, occupies about one third of the plasma membrane lipid content and is an essential component of membrane in animal cells [34, 35]. High level of cholesterol is cytotoxic, therefore should be tightly regulated. Multiple mechanisms are working, regulating cholesterol uptake, synthesis and metabolism as well as efflux system operating for the maintenance of the optimal intracellular cholesterol concentration [35]. Nevertheless, this regulation is often dysregulated in cancer, potentially contributing to hallmarks of cancer including growth, anti-apoptosis and resistance to chemotherapy [36]. Moreover, cholesterol is rich in cancer cell membranes and has been linked to reduced sensitivity to platinum-based chemotherapy in lung adenocarcinoma [12]. Likewise, clinical significance of cholesterol in ovarian cancer has been suggested by many recent studies. The long-term use of statin derivatives, inhibitor of cholesterol synthesis, has been shown to be associated with reduced risk of ovarian cancer development [23–25, 37] and improved survival for patients with ovarian cancer [38]. In in vitro studies, statins significantly reduces transporter activity of ABCG2 or MDR1 but did not expression of mRNA, through depletion of cellular cholesterol [21]. The transport functions of both ABCG2 and MDR1 are significantly affected by cellular and membrane cholesterol levels [11, 22, 39–41]. These results and our results have prompted us to hypothesize that cholesterol enriched in malignant ascites contributes to acquire chemoresistance in ovarian cancer through upregulation of ABCG2 or MDR1 protein. Consistent with previous study, our data reveal that cholesterol alone and malignant ascites derived from ovarian cancer patients upregulates the protein expression of ABCG2 and MDR1, associated with poor prognosis and chemoresistance in ovarian cancer patients [42–44].

Response to cholesterol treatment was variable among the three ovarian cancer cell lines we used in this study. We reasoned that the observed differences are partly due to the differences in cell lines themselves. PA-1 cell has been previously shown to be more sensitive to chemotherapeutics [45]. Cell line difference is hard to define. In the present study, we found that ABCG2, MDR1 and LXRα/β protein expression levels were correlated with resistance to CDDP and PAC in three ovarian cancer cells (Fig. 1 and Fig. 3). Furthermore, PA-1 exhibited relatively lower LXRα/β expression levels but increased expression in response to cholesterol treatment (Fig. 3a and c). Although our data is limited with three ovarian cancer cell lines, these findings indicate that the cholesterol treatment may enhance resistance in ovarian cancer cells through increased LXRα/β expression.

Cholesterol influences the expression of ABC transporters by regulating their gene expression via nuclear receptors (NRs). The NR superfamily comprises 48 members in the human genome and represents the largest currently known family of transcription factors [22]. To date, several NRs involved in the recognition of lipid ligands have been shown to affect the gene expression of human ABCB1 and ABCG2 transport function causing multidrug resistance [46–48]. Likewise, cholesterol and malignant ascites treatment upregulated cholesterol receptor, LXRα/β expression. Upregulation of MDR1 expression driven by cholesterol was significantly reversed by silencing LXRα/β in PA-1 cells pre-treated with cholesterol, compared with that of untreated cells. Interestingly, silencing LXRα/β did not significantly reduce the expression of ABCG2 in PA-1 cells (Fig. 4b). Notably, silencing LXRα/β, significantly reduced ovarian cancer cell response to cholesterol induced resistance to CDDP and PAC (Fig. 4c). It is also possible that stress signaling transduction pathway transcriptionally activate ABCG2 expression [46]. In the case of malignant ascites treatment, our group previously reported elevated levels of interleukin 6 (IL-6) in malignant ascites [6] and it has been reported that IL-6 stimulates ABCG2 expression [49]. To explain these differences, we postulate that there is another regulatory factor induced by both cholesterol and malignant ascites which may indirectly activate ABCG2 expression, independent of LXRα/β, a topic for future studies. Our results imply that inhibition of MDR1 alone reverses acquired chemoresistance in ovarian cancer.

## Conclusions

To date, no study has investigated a direct effect of cholesterol on acquired chemoresistance in ovarian cancer. The results of our research is novel in suggesting that cholesterol enriched in malignant ascites contributes to poor prognosis by driving the expression of these ABC transporters and confer chemoresistance in ovarian cancer cells. As for the mechanism(s) by which cholesterol levels are elevated in ascites, it needs more studies. Collectively, our study underscores the importance of future studies to investigate the mechanism(s) by which cholesterol levels are elevated in ascites and how to manage these in clinical setting.

## Additional files

Additional file 1: **Figure S1.** Cholesterol loading and ovarian cancer cell viability. PA-1, OVCAR-3 and SKOV-3 ovarian cancer cell lines treated with indicated concentration of water soluble cholesterol (**A**) and cholesterol loading control, MβCD (**B**) for 48 h. Cell viability were measured using MTT assay. *P < 0.05, **P < 0.01 and ***P < 0.001. **Figure S2.** Cholesterol increase CDDP and PAC resistance in ovarian cancer cells. (**A-B**) The indicated concentrations of CDDP and PAC were treated to three ovarian cancer cell lines (PA-1, OVCAR-3 and SKOV-3) and ascites derived ovarian cancer cells (A8, A39 and A53) with or without cholesterol (5 μg/ml) pre-treatment for 24 h. Cell viability was determined by MTT assay. **Figure S3.** Cholesterol in ovarian cancer patient derived ascites promotes resistance to CDDP and PAC combination. (**A**) The indicated combination concentration of CDDP and PAC were treated to PA-1 cell. Cell viability was determined by MTT assay. (**B1**) CDDP and PAC combination induced apoptotic cell death determined by Annexin V/PI staining for 24 h after treatment with each respective malignant ascites. (Black box indicate control without CDDP+PAC combination treatment). (**B2**) Correlation between cholesterol levels in malignant ascites and relative ratio o number of CDDP and PAC combination induced apoptotic cell death. The correlation coefficient square (R^2^) was determined by Pearson’s correlation coefficient test. Significant differences are indicated as follows. *P < 0.05, **P < 0.01. (DOCX 335 kb)

Additional file 2: **Table S1.** Information of primary cancer cells isolated from ovarian cancer patient derived ascites. (DOCX 14 kb)

Additional file 3: **Table S2.** Description of patients with malignant ascites (DOCX 12 kb)

Additional file 4: **Table S3.** Description of patients with non-malignant ascites. Data not shown. ROC curve and Youden index analysis to determine the cut-off value for ascites cholesterol. (A) ROC curve (B) Youden index analysis. (DOCX 13 kb)

## Abbreviations

CDDP: Cisplatin
CHO: Cholesterol
LXRα/β: Liver x receptor α/β
MβCD: Methyl beta cyclodextrin
NRs: Nuclear receptors
PAC: Paclitaxel
RFS: Recurrence free survival
SACG: Serum ascites cholesterol gradient
