# Supplementary figures and images for: Activation of LXRɑ/β by cholesterol in malignant ascites promotes chemoresistance in ovarian cancer
Source: BMC Cancer. 2018 Dec 10;18:1232. doi: 10.1186/s12885-018-5152-5 (PMC6288854; doi:10.1186/s12885-018-5152-5)

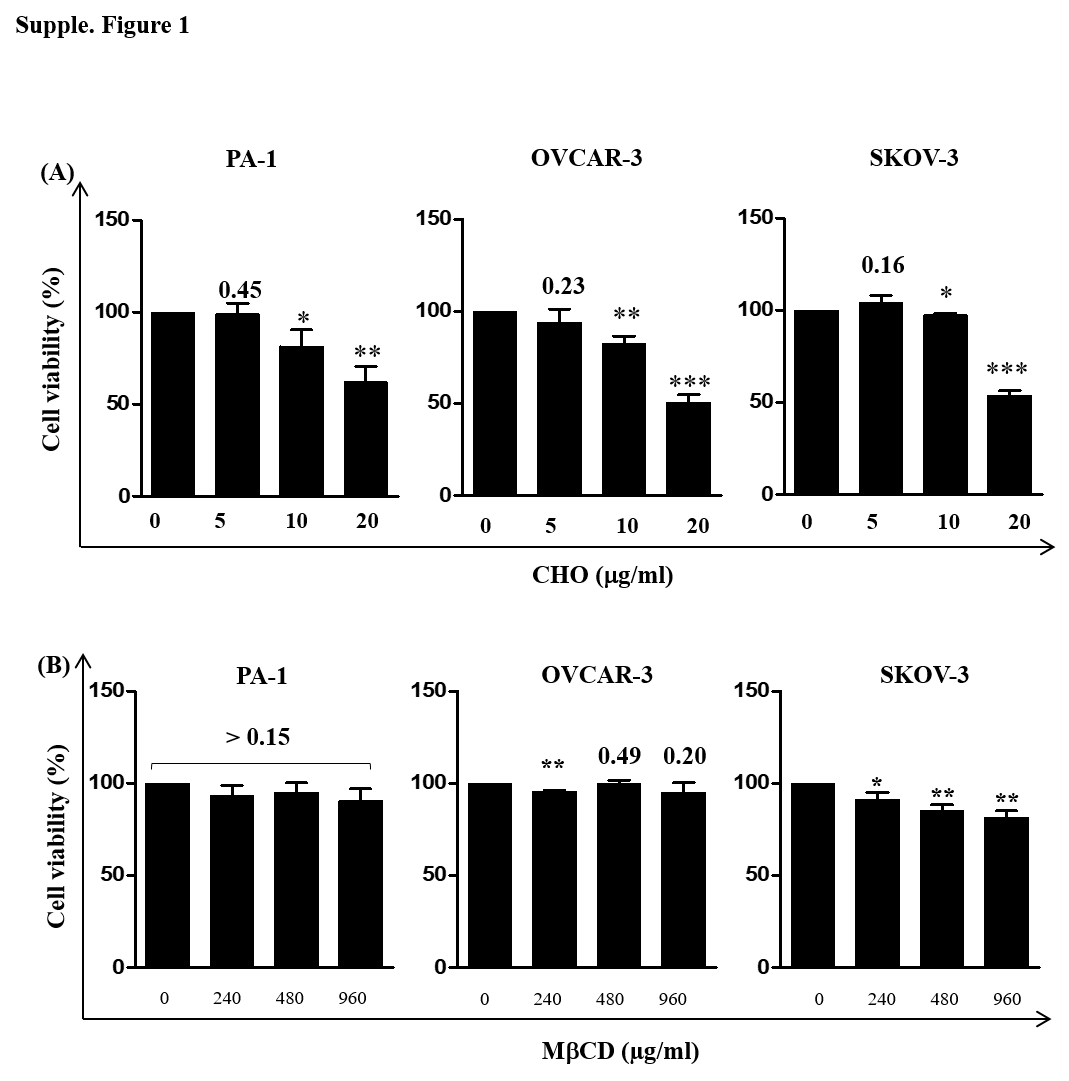


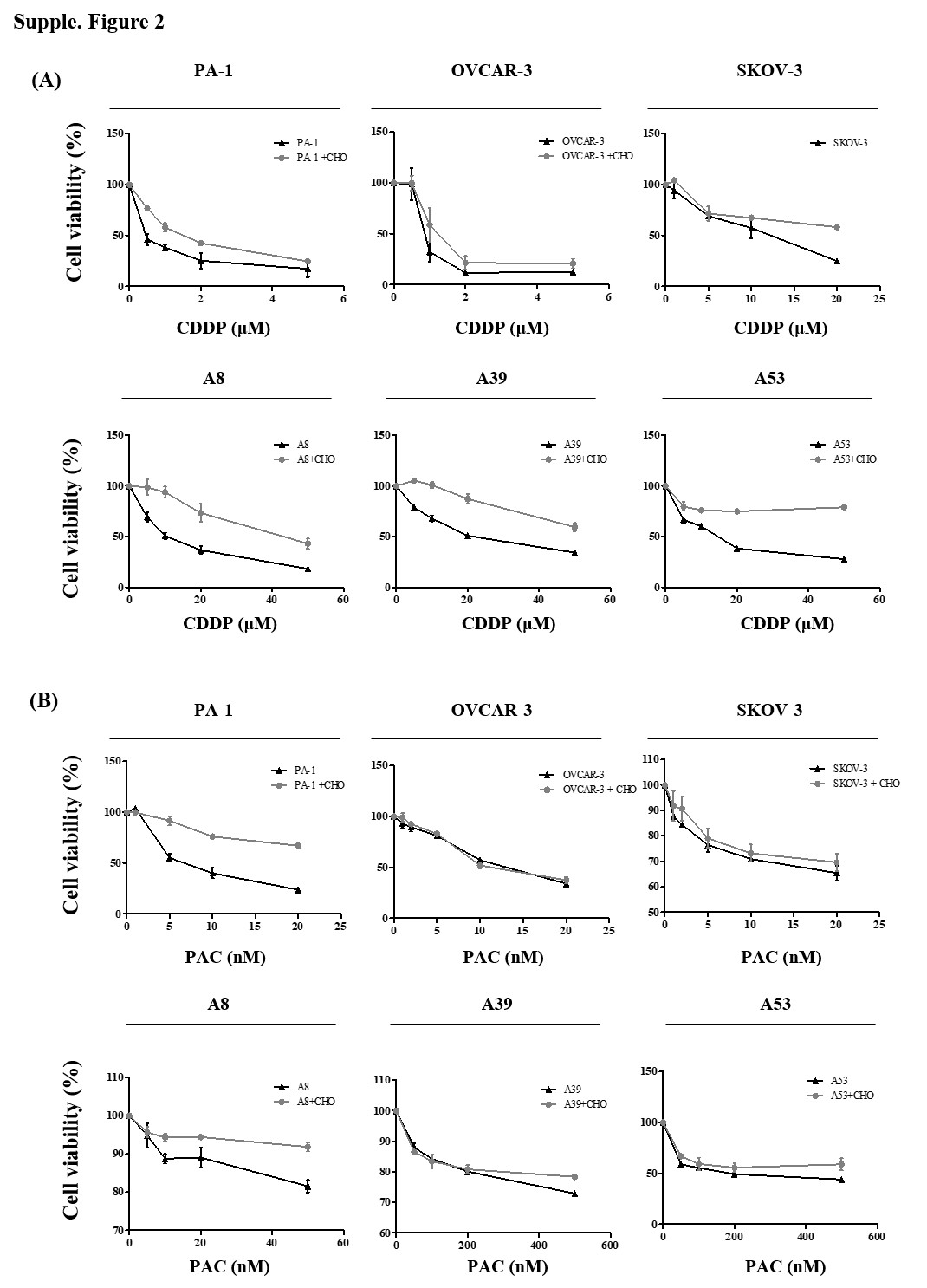


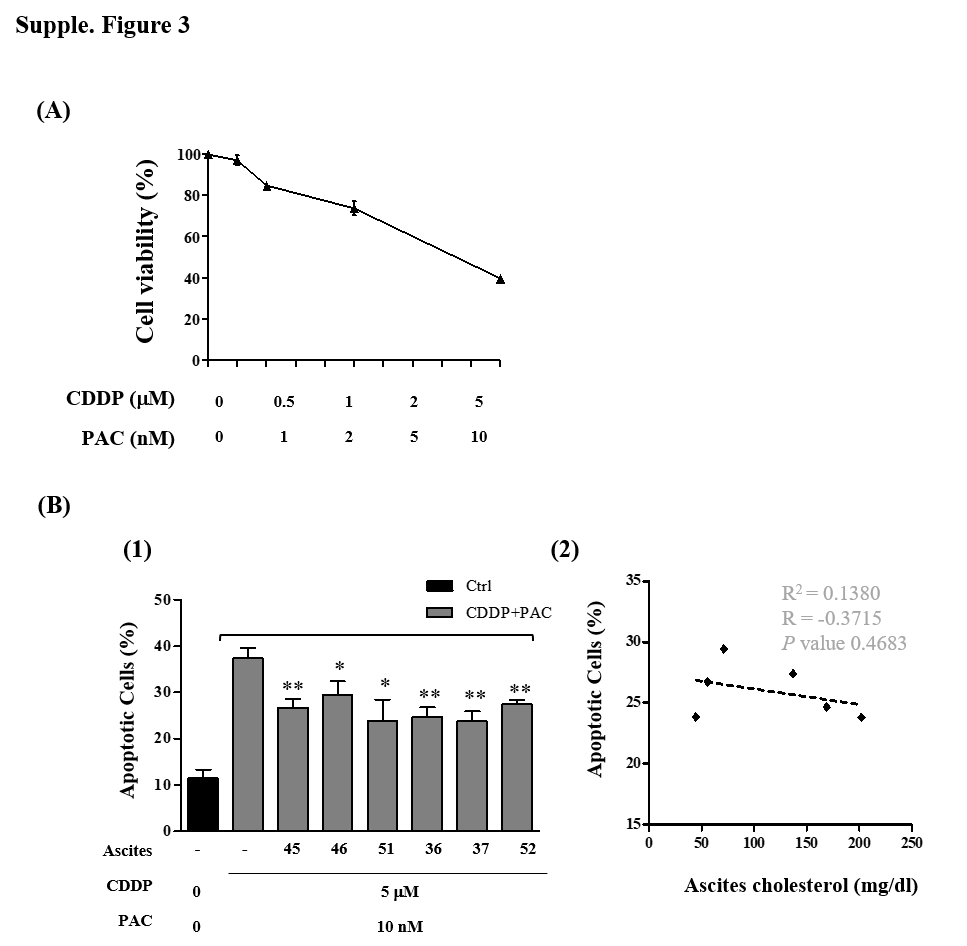

Supplement: Supplementary file 1 — Figure S1. Cholesterol loading and ovarian cancer cell viability. PA-1, OVCAR-3 and SKOV-3 ovarian cancer cell lines treated with indicated concentration of water soluble cholesterol (A) and cholesterol loading control, MβCD (B) for 48 h. Cell viability were measured using MTT assay. *P < 0.05, **P < 0.01 and ***P < 0.001. Figure S2. Cholesterol increase CDDP and PAC resistance in ovarian cancer cells. (A-B) The indicated concentrations of CDDP and PAC were treated to three ovarian cancer cell lines (PA-1, OVCAR-3 and SKOV-3) and ascites derived ovarian cancer cells (A8, A39 and A53) with or without cholesterol (5 μg/ml) pre-treatment for 24 h. Cell viability was determined by MTT assay. Figure S3. Cholesterol in ovarian cancer patient derived ascites promotes resistance to CDDP and PAC combination. (A) The indicated combination concentration of CDDP and PAC were treated to PA-1 cell. Cell viability was determined by MTT assay. (B1) CDDP and PAC combination induced apoptotic cell death determined by Annexin V/PI staining for 24 h after treatment with each respective malignant ascites. (Black box indicate control without CDDP+PAC combination treatment). (B2) Correlation between cholesterol levels in malignant ascites and relative ratio o number of CDDP and PAC combination induced apoptotic cell death. The correlation coefficient square (R2) was determined by Pearson’s correlation coefficient test. Significant differences are indicated as follows. *P < 0.05, **P < 0.01. (DOCX 335 kb) [file 12885_2018_5152_MOESM1_ESM.docx]
