# Supplementary material for: Activation of LXRɑ/β by cholesterol in malignant ascites promotes chemoresistance in ovarian cancer
Source: BMC Cancer. 2018 Dec 10;18:1232. doi: 10.1186/s12885-018-5152-5 (PMC6288854; doi:10.1186/s12885-018-5152-5)
Supplement: Supplementary file 2 — Table S1. Information of primary cancer cells isolated from ovarian cancer patient derived ascites. (DOCX 14 kb) [file 12885_2018_5152_MOESM2_ESM.docx]

| Ascites Patient # | Histological type | Stage |
| --- | --- | --- |
| 8 | Serous | IIIC |
| 25 | Serous | IV |
| 32 | Clear cell | IIC |
| 34 | Serous | IVB |
| 37 | Serous | IIIC |
| 38 | Endometrioid | IVA |
| 39 | Clear cell | IIIC |
| 42 | Serous | IIIC |
| 46 | Serous | IV |
| 47 | Serous | IV |
| 48 | Serous | IV |
| 50 | Serous | IIIC |
| 51 | Serous | IVB |
| 53 | Serous | IVB |
