# Supplementary material for: Activation of LXRɑ/β by cholesterol in malignant ascites promotes chemoresistance in ovarian cancer
Source: BMC Cancer. 2018 Dec 10;18:1232. doi: 10.1186/s12885-018-5152-5 (PMC6288854; doi:10.1186/s12885-018-5152-5)
Supplement: Supplementary file 3 — Table S2. Description of patients with malignant ascites (DOCX 12 kb) [file 12885_2018_5152_MOESM3_ESM.docx]

| **Variables** | | **N(%) N=45** |
| --- | --- | --- |
| **Age** | Mean± SD | 56.6 ±10.6 |
| **Stage** | I  II  III  IV | 5(11.1)  4(8.9)  20(44.4)  16(35.6) |
| **Histology** | Serous  Mucinous  Clear  Mixed | 30 (66.7)  6 (13.3)  6 (13.3)  3 (6.7) |
| **Grade** | 1-2  3  Unknown | 8(17.8)  28(62.2)  9(20.0) |
| **Neoadjuvant Chemotherapy** | Yes | 12(26.7) |
| **CA125** | Median (range) | 876 (4.4-15700) |
| **Ascites cytology** | Positive | 45(100) |
| **Recurrence** | Yes | 23 (51.1) |
