# Supplementary material for: Activation of LXRɑ/β by cholesterol in malignant ascites promotes chemoresistance in ovarian cancer
Source: BMC Cancer. 2018 Dec 10;18:1232. doi: 10.1186/s12885-018-5152-5 (PMC6288854; doi:10.1186/s12885-018-5152-5)
Supplement: Supplementary file 4 — Table S3. Description of patients with non-malignant ascites. Data not shown. ROC curve and Youden index analysis to determine the cut-off value for ascites cholesterol. (A) ROC curve (B) Youden index analysis. (DOCX 13 kb) [file 12885_2018_5152_MOESM4_ESM.docx]

| **Patient ID** | **Disease** | **Age range** |
| --- | --- | --- |
| **N1** | Paratubal cyst | 39-76 |
| **N2** | Mucinous cystadenoma |  |
| **N3** | Teratoma |  |
